# Supplementary material for: ORBIT: Annotation-Aware Empirical Enrichment and Semantic Reranking for Interpretable Functional-Class Recovery
Source: bioRxiv. 2026 Jul 7:2026.07.01.735870. Preprint. [Version 1] doi: 10.64898/2026.07.01.735870 (PMC13370979; doi:10.64898/2026.07.01.735870)
Supplement: Supplement 1 [file media-1.pdf]

SUPPLEMENTARY INFORMATION

ORBIT: Annotation-Aware Empirical Enrichment and Semantic Reranking for Interpretable Functional-Class Recovery

Benjamin L. Kidder[1-2*]

[1]Department of Oncology, Wayne State University School of Medicine, Detroit, MI, USA

[2]Karmanos Cancer Institute, Wayne State University School of Medicine, Detroit, MI, USA

Running title: ORBIT semantic gene-set interpretation

*Correspondence:

Benjamin L. Kidder

Email : benjamin.kidder@wayne.edu

Supplemental Figures

Figure S1. Benchmark robustness analyses. (A) Mean reciprocal rank stratified by benchmark tier. Error bars show bootstrap 95% confidence intervals, and within-bar labels indicate the number of benchmark gene sets in each tier. (B) Expanded paired ORBIT advantage analysis across MRR and top-k recovery metrics. Points show mean paired deltas relative to the indicated external baseline, horizontal bars show bootstrap 95% confidence intervals, and p-values are from paired sign-flip permutation tests.

Figure S2. Benchmark summary across ORBIT modes and external baselines. (A) Core benchmark performance across MRR and top-k recovery metrics for ORBIT semantic, ORBIT classical ORA, ORBIT empirical, Enrichr Gene Ontology molecular-function, PANTHER Gene Ontology molecular-function, and PANTHER protein-class baselines. (B) Exploratory benchmark best-rank counts across methods. (C) Core benchmark MRR by benchmark tier across methods.

Figure S3. Expanded external enrichment comparator analysis. (A) Mean reciprocal-rank recovery for ORBIT, Enrichr, PANTHER, and g:Profiler comparator outputs on the core and exploratory benchmark sets. (B) Paired core-benchmark MRR advantage for ORBIT relative to each external comparator, with bootstrap 95% confidence intervals. (C) Core benchmark top-k recovery rates for the same comparator set.

Figure S4. Semantic-reduction audit for ORBIT redundancy control. (A) Number of significant enriched terms before and after semantic representative selection in the GPCR mixed-hard case study. (B) Largest semantic neighborhoods collapsed by ORBIT into representative terms.

Figure S5. PBMC clustered cell-type by function-class heatmap. Marker-gene function-class fractions are shown for each Seurat-annotated PBMC cell type. Values indicate the fraction of marker genes assigned to each ORBIT function class, highlighting broad cell-type-specific annotation patterns that complement the enrichment dot plot in Figure 4.

Figure S6. PBMC marker-gene effect-size and prevalence plot. Top marker genes for each annotated PBMC cell type are shown by rank within cell type. Color indicates average log2 fold-change, and point size indicates marker prevalence or supporting-cell fraction, depending on the plotted variable. Marker labels identify genes supporting the cell-type annotation patterns summarized in Figure 4 and Supplementary Figure S5.

A

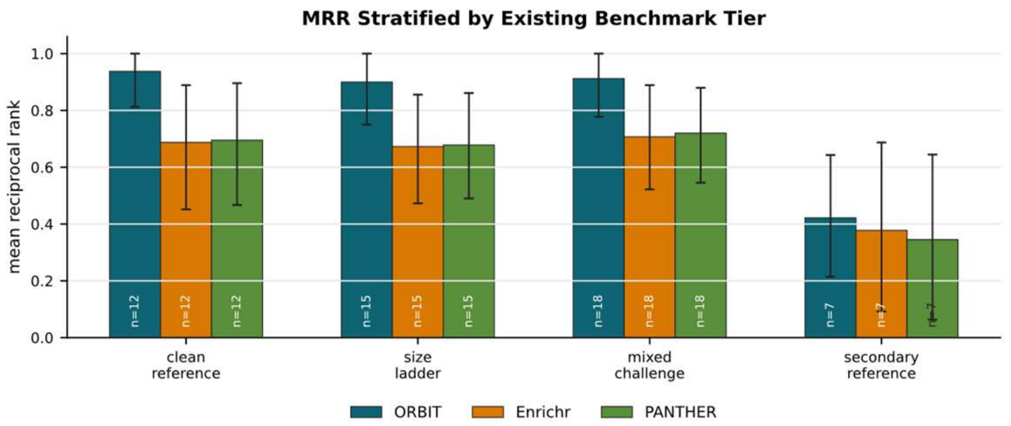

MRR Stratified by Existing Benchmark Tier
mean reciprocal rank
1.0
0.8
0.6
0.4
0.2
0.0
n=12
n=15
n=18
n=7
clean reference
size ladder
mixed challenge
secondary reference
ORBIT
Enrichr
PANTHER

B

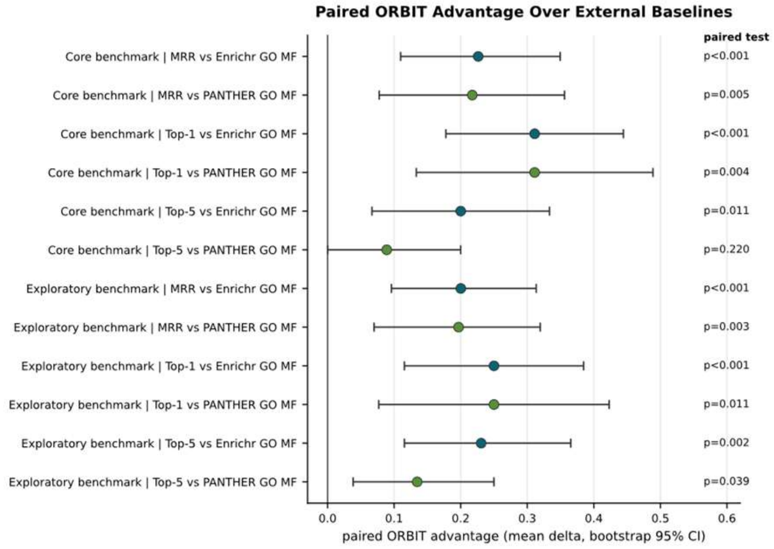

Paired ORBIT Advantage Over External Baselines
paired test
Core benchmark | MRR vs Enrichr GO MF
p<0.001
Core benchmark | MRR vs PANTHER GO MF
p=0.005
Core benchmark | Top-1 vs Enrichr GO MF
p<0.001
Core benchmark | Top-1 vs PANTHER GO MF
p=0.004
Core benchmark | Top-5 vs Enrichr GO MF
p=0.011
Core benchmark | Top-5 vs PANTHER GO MF
p=0.220
Exploratory benchmark | MRR vs Enrichr GO MF
p<0.001
Exploratory benchmark | MRR vs PANTHER GO MF
p=0.003
Exploratory benchmark | Top-1 vs Enrichr GO MF
p<0.001
Exploratory benchmark | Top-1 vs PANTHER GO MF
p=0.011
Exploratory benchmark | Top-5 vs Enrichr GO MF
p=0.002
Exploratory benchmark | Top-5 vs PANTHER GO MF
p=0.039
0.0 0.1 0.2 0.3 0.4 0.5 0.6
paired ORBIT advantage (mean delta, bootstrap 95% CI)

Figure S1

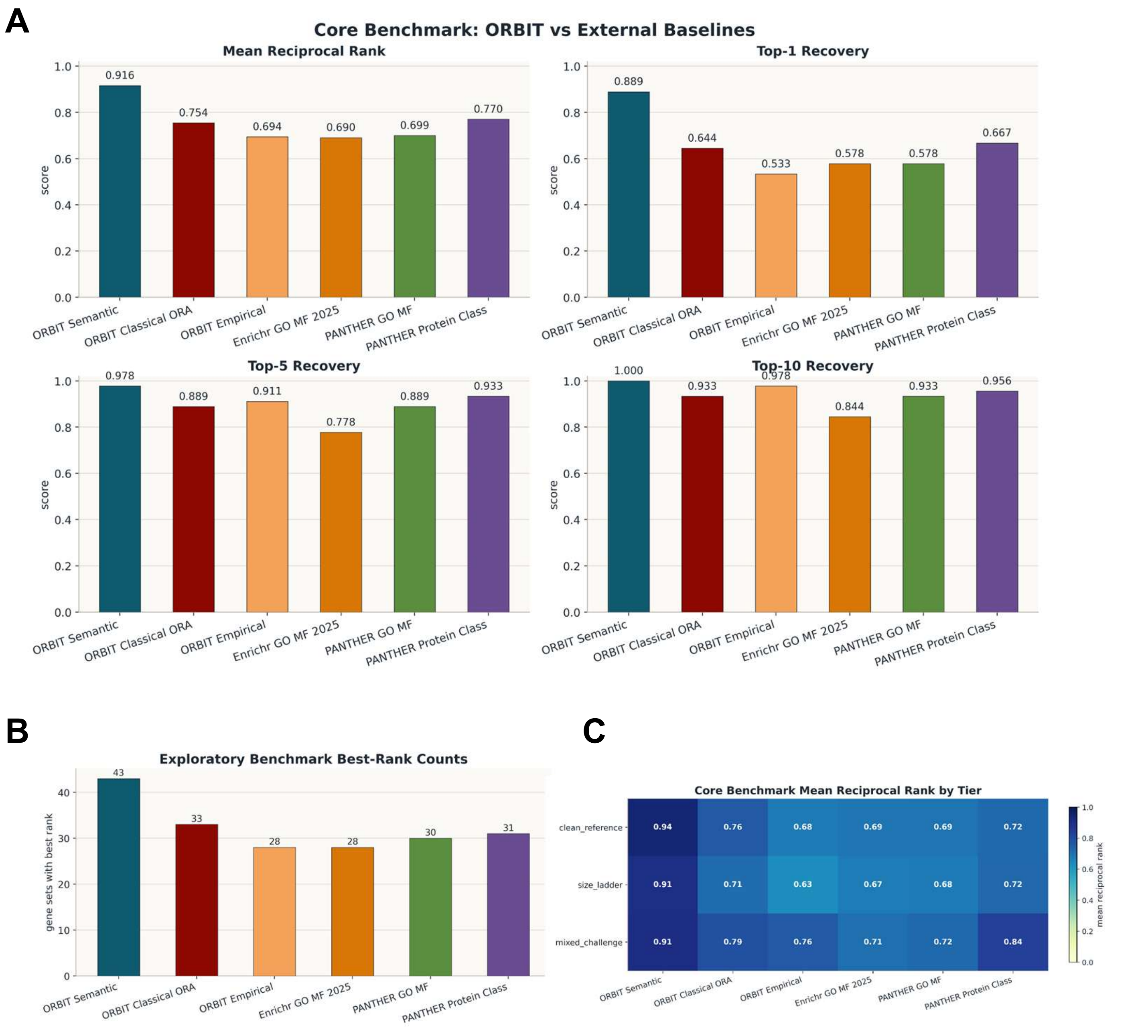
A
Core Benchmark: ORBIT vs External Baselines
Mean Reciprocal Rank
0.916
0.754
0.694
0.690
0.699
0.770
Top-1 Recovery
0.889
0.644
0.533
0.578
0.578
0.667
Top-5 Recovery
0.978
0.889
0.911
0.778
0.889
0.933
Top-10 Recovery
1.000
0.933
0.978
0.844
0.933
0.956
score
ORBIT Semantic
ORBIT Classical ORA
ORBIT Empirical
Enrichr GO MF 2025
PANTHER GO MF
PANTHER Protein Class
B
Exploratory Benchmark Best-Rank Counts
gene sets with best rank
43
33
28
28
30
31
C
Core Benchmark Mean Reciprocal Rank by Tier
clean_reference
size_ladder
mixed_challenge
0.94
0.76
0.68
0.69
0.69
0.72
0.91
0.71
0.63
0.67
0.68
0.72
0.91
0.79
0.76
0.71
0.72
0.84
mean reciprocal rank

Figure S2

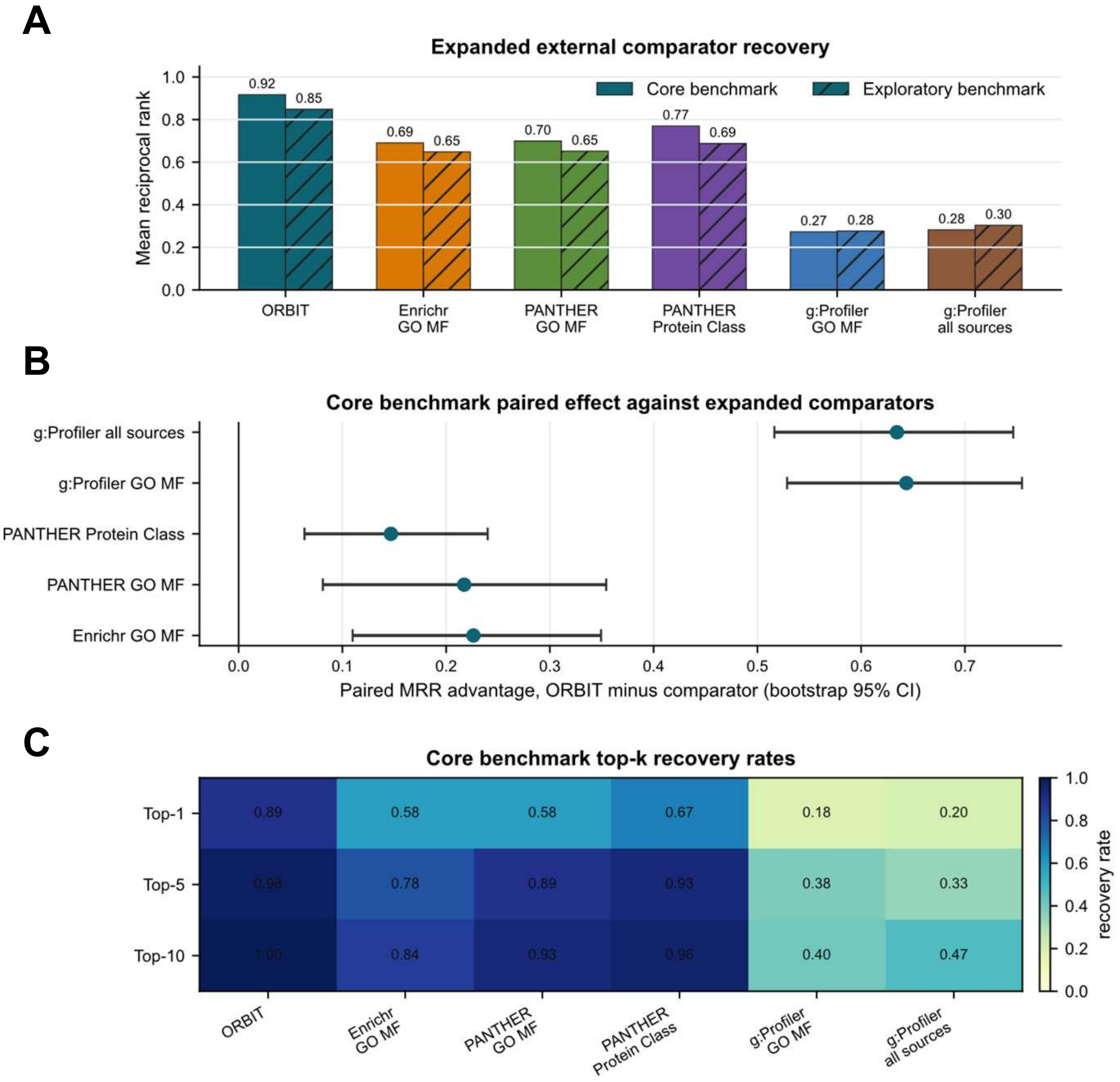
A
Expanded external comparator recovery
Mean reciprocal rank
Core benchmark
Exploratory benchmark
0.92
0.85
0.69
0.65
0.70
0.65
0.77
0.69
0.27
0.28
0.28
0.30
ORBIT
Enrichr GO MF
PANTHER GO MF
PANTHER Protein Class
g:Profiler GO MF
g:Profiler all sources
B
Core benchmark paired effect against expanded comparators
g:Profiler all sources
g:Profiler GO MF
PANTHER Protein Class
PANTHER GO MF
Enrichr GO MF
Paired MRR advantage, ORBIT minus comparator (bootstrap 95% CI)
C
Core benchmark top-k recovery rates
Top-1
Top-5
Top-10
0.89
0.58
0.58
0.67
0.18
0.20
0.98
0.78
0.89
0.93
0.38
0.33
1.00
0.84
0.93
0.96
0.40
0.47
recovery rate
ORBIT
Enrichr GO MF
PANTHER GO MF
PANTHER Protein Class
g:Profiler GO MF
g:Profiler all sources

Figure S3

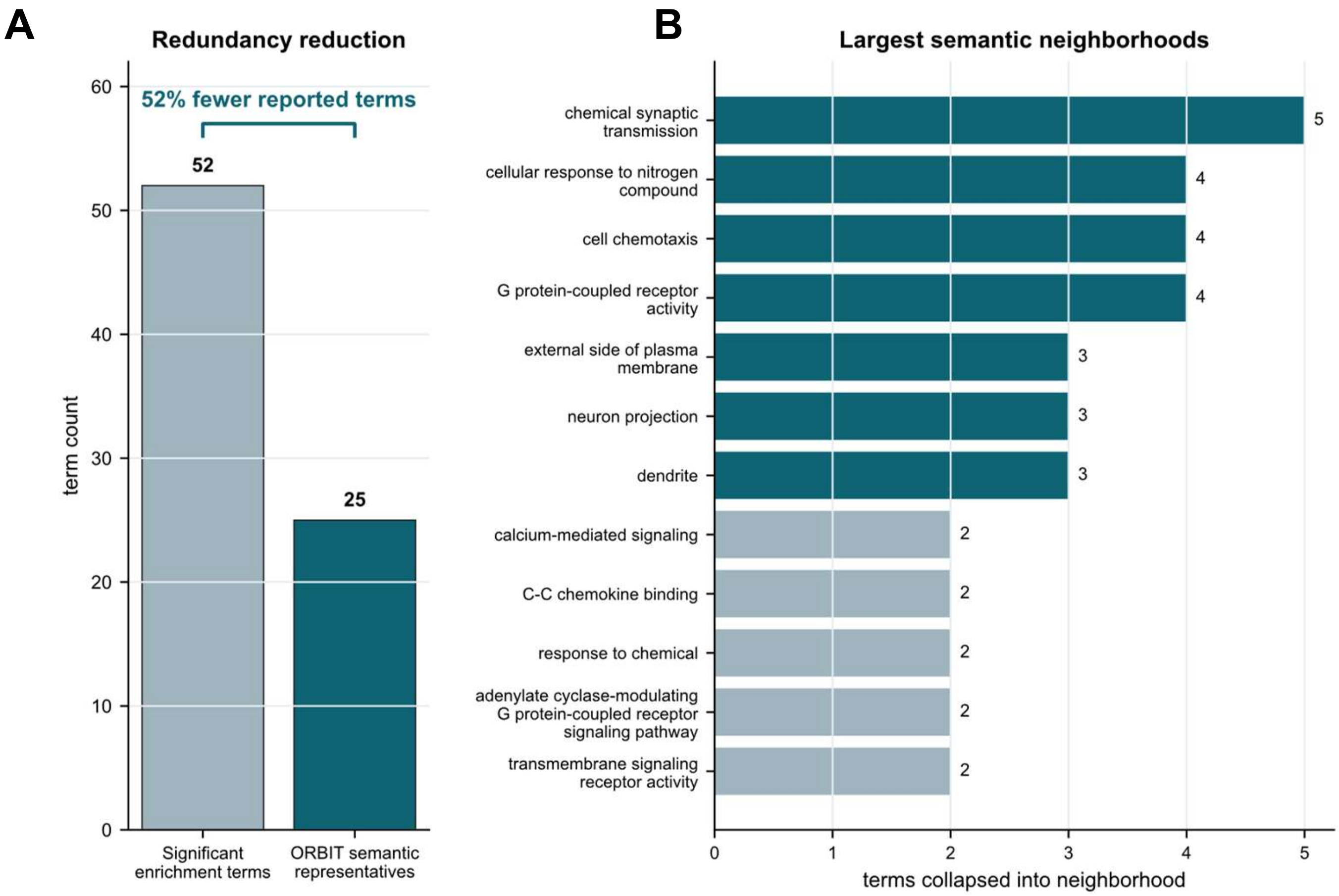
A
Redundancy reduction
52% fewer reported terms
52
25
term count
0
10
20
30
40
50
60
Significant enrichment terms
ORBIT semantic representatives
B
Largest semantic neighborhoods
chemical synaptic transmission
cellular response to nitrogen compound
cell chemotaxis
G protein-coupled receptor activity
external side of plasma membrane
neuron projection
dendrite
calcium-mediated signaling
C-C chemokine binding
response to chemical
adenylate cyclase-modulating G protein-coupled receptor signaling pathway
transmembrane signaling receptor activity
5
4
4
4
3
3
3
2
2
2
2
2
0
1
2
3
4
5
terms collapsed into neighborhood

Figure S4

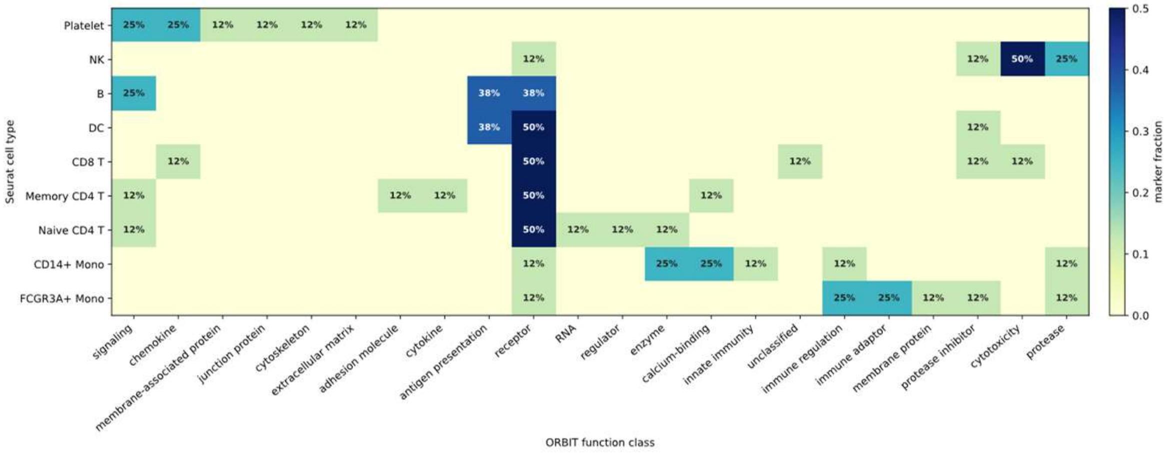

Platelet
NK
B
DC
CD8 T
Memory CD4 T
Naive CD4 T
CD14+ Mono
FCGR3A+ Mono
Seurat cell type
signaling
chemokine
membrane-associated protein
junction protein
cytoskeleton
extracellular matrix
adhesion molecule
cytokine
antigen presentation
receptor
RNA
regulator
enzyme
calcium-binding
innate immunity
unclassified
immune regulation
immune adaptor
membrane protein
protease inhibitor
cytotoxicity
protease
ORBIT function class
marker fraction
0.0
0.1
0.2
0.3
0.4
0.5

Figure S5

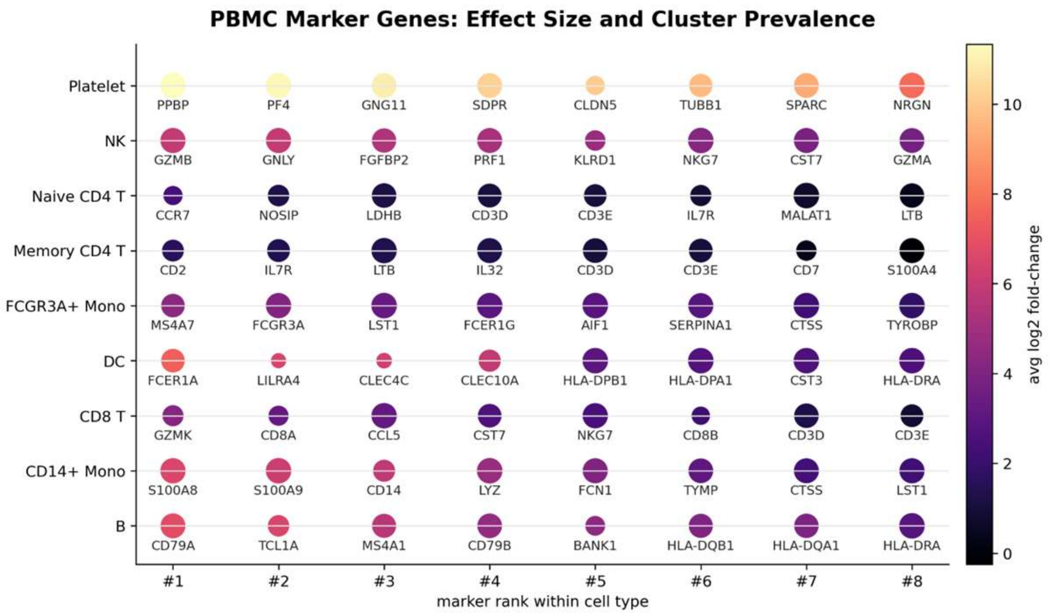

PBMC Marker Genes: Effect Size and Cluster Prevalence
Platelet
NK
Naive CD4 T
Memory CD4 T
FCGR3A+ Mono
DC
CD8 T
CD14+ Mono
B
PPBP PF4 GNG11 SDPR CLDN5 TUBB1 SPARC NRGN
GZMB GNLY FGFBP2 PRF1 KLRD1 NKG7 CST7 GZMA
CCR7 NOSIP LDHB CD3D CD3E IL7R MALAT1 LTB
CD2 IL7R LTB IL32 CD3D CD3E CD7 S100A4
MS4A7 FCGR3A LST1 FCER1G AIF1 SERPINA1 CTSS TYROBP
FCER1A LILRA4 CLEC4C CLEC10A HLA-DPB1 HLA-DPA1 CST3 HLA-DRA
GZMK CD8A CCL5 CST7 NKG7 CD8B CD3D CD3E
S100A8 S100A9 CD14 LYZ FCN1 TYMP CTSS LST1
CD79A TCL1A MS4A1 CD79B BANK1 HLA-DQB1 HLA-DQA1 HLA-DRA
#1 #2 #3 #4 #5 #6 #7 #8
marker rank within cell type
avg log2 fold-change
0 2 4 6 8 10

Figure S6
